# Effects of exercise supplementary to standard therapy on cognition and sleep in depression: a randomised controlled trial

**DOI:** 10.3389/fpsyt.2026.1650334

**Published:** 2026-02-03

**Authors:** Cong Liu, Fei Zhai, Min Li, Huiying Wang, Jianhong Zhang, Ziyang Ji, Hengfen Li

**Affiliations:** 1 The First Affiliated Hospital of Zhengzhou University, Zhengzhou, China; 2 The Affiliated People's Hospital of Xinxiang Medical University, Xinxiang, China; 3 The Second Affiliated Hospital of Xinxiang Medical University, Xinxiang, China; 4 Henan Collaborative Innovation Center of Prevention and Treatment of Mental Disorder, Xinxiang, China; 5 Brain Institute, Henan Academy of Innovations in Medical Science, Xinxiang, China

**Keywords:** major depression, randomised controlled trial, exercise therapy, executive function, sleep quality

## Abstract

**Purpose:**

To explore the Effects of Exercise Supplementary to Standard Therapy on Cognition and Sleep in Depression.

**Methods:**

We randomized 273 inpatients with first-episode severe depression, 234 completed 6 weeks, conventional treatment, conventional treatment combined with aerobic exercises and conventional treatment combined with stretching or resistance training exercises group. Hamilton Depression Scale-24 (HAMD24), Pittsburgh Sleep Quality Index (PSQI), Montreal Cognitive Assessment (MOCA), Chinese Version of the Trail-Making Test (C-TMT), and Stroop Color and Word Test (SCWT) were used to evaluate the patients respectively before and after intervention. The primary analysis estimated was the between-group difference in post-treatment scores at 6 weeks under randomized allocation (post-status estimated); within-group changes were summarized descriptively.

**Results:**

After intervention, HAMD24, PSQI, C-TMT-A, and C-TMT-B scores of patients in each group were all lower than those before intervention. HAMD24, PSQI, and C-TMT-A scores of patients in Groups B and C showed lower than those of Group A. C-TMT-B score of patients in Group B was lower than that of Group C, and the score of Group C was lower than that of Group A. MOCA, SCWT scores of patients in each group were higher than those before intervention. Stroop Word and Stroop Color scores were significantly higher in Groups B and C than in Group A. MOCA and Stroop Color-Word scores of patients in Group B were higher than those of Group C. However, scores of Group C were higher than those of Group A.

**Conclusion:**

Both aerobic exercises and stretching or resistance training exercises as adjuncts to conventional treatment improved depressive symptoms, sleep quality, and cognitive function in patients with first-episode severe depression. Patterns were consistent with greater improvement in select executive-function measures in the aerobic arm; confirmation with baseline-adjusted analyses is warranted.

## Introduction

Depression is a mental illness characterized by a persistent low mood and reduced interest and energy levels, significantly impairing the quality of life (1, 2). The global prevalence of depression is approximately 5% (3). This condition profoundly impacts the physical and mental health of patients while also imposing substantial social and economic burdens. By 2030, depression is anticipated to become the leading cause of disease burden globally (4). Consequently, research on depression treatment remains a critical focus in the field of global health.

In clinical practice, depression is commonly associated with low mood, slow thinking, and reduced volitional activity. However, patients with depression often also experience varying degrees of sleep disorders, cognitive dysfunction, and executive dysfunction. Sleep disorders, which are one of the primary symptoms of depression, serve as a significant risk factor for the onset and progression of the condition (5). Sleep disorders and depression frequently co-occur, demonstrating a bidirectional relationship. Approximately 80-90% of individuals with depression report sleep disturbances (6, 7), which, in turn, can lead to negative emotions such as anxiety and tension, thereby exacerbating depression (6). Cognitive dysfunction is another critical symptom prevalent among patients with depression (8, 9). Even after depressive symptoms are alleviated, cognitive impairments may persist, affecting or limiting the patient's functional abilities in various areas and severely impacting their quality of life (10, 11). Furthermore, cognitive dysfunction can increase the risk of recurrent depression and reduce patient adherence to medication regimens (12). Therefore, it is essential to address not only the emotional symptoms of depression but also the cognitive impairments that may continue even after the depressive symptoms have improved. Executive function (13) is a subset of cognitive function involving the regulation and control of other cognitive processes necessary for completing complex tasks. It encompasses several key functions, including planning, judgment, decision-making, inhibition of inappropriate responses, and initiating and controlling goal-directed behaviours. This complex cognitive activity is significantly impacted by depression (14). Consequently, treatment for depression should not only address the patients' emotional symptoms but also their sleep quality, cognitive function, and particularly their executive function.

With the continuous advancement and development of medical science, the treatment options for severe depression have become increasingly diverse. However, they remain largely confined to drug therapy and psychological therapy. Although antidepressants are more effective than placebos and are widely used in clinical practice (15), only approximately 50% of patients with severe depression experience significant clinical improvement after taking these medications (16). Furthermore, issues such as stigma, side effects, economic burden, and potential addiction often lead to poor treatment adherence and interruptions, resulting in fluctuations or even worsening of the depression. In addition to medication, psychotherapy is also recommended as a first-line treatment in many depression guidelines (17–19). Nevertheless, the widespread implementation of psychological treatment is challenging due to its specialized nature, lengthy duration, and considerable economic burden. Therefore, it is crucial to explore new (combination) treatment methods to better address the needs of patients with severe depression.

In recent years, there has been increasing attention on the study of exercise therapy for depression (20–22). Both aerobic exercises and stretching or resistance training exercises have been shown to be effective in treating depression (23–29). However, there is limited research comparing the effects of stretching or resistance training exercises with aerobic exercises in the treatment of severe depression. To address this gap, we incorporated interventions of aerobic exercises therapy, stretching or resistance training exercises therapy, and non-exercise therapy into the conventional treatment for patients with severe depression. We conducted a comparative analysis of their clinical efficacy, aiming to provide a reference for developing exercises therapy prescriptions for patients with severe depression. In our research, the assessment of clinical efficacy not only includes the evaluation of depressive symptoms and sleep quality, but also encompasses the assessment of cognitive function, for exercise can be understood as a form of energetic mobilisation that, through cyclic engagement and recovery, promotes the restoration of attentional resources (30). While prior studies (31, 32) have explored exercise as an add-on treatment for depression, their focus has predominantly been on mild-to-moderate cases or lacked direct comparisons between aerobic exercises and stretching or resistance training exercises modalities. This study uniquely targets inpatients with severe depression, providing head-to-head comparisons of these exercise types alongside conventional treatment. In order to reduce the duration of depression and the impact of previous treatment, patients with first episode of severe depressive disorder were selected as the study subjects.

## Materials and methods

### Participants

#### Inclusion criteria

- Age 18-60 years, with no gender limitations.
- Diagnosed with depression (first episode) according to The International Statistical Classification of Diseases and Related Health Problems 10th Revision (ICD-10).
- Hamilton Depression Scale-24 (HAMD24) score greater than 35.
- Treated with a first-line antidepressant, selective serotonin reuptake inhibitor (SSRI), as monotherapy according to depression treatment guidelines (33–35).
- No visual or auditory impairments, capable of completing scale assessments and other tests. f) Educational level of junior high school or above.

#### Exclusion criteria

- History of manic episodes.
- Presence of organic brain disease or significant physical illness.
- Dependence on psychoactive substances.
- Scheduled for electroconvulsive therapy.
- Pregnant or lactating.
- Presence of psychotic symptoms.
- Inability to engage in exercise.
- Colour blindness or colour weakness.
- Patients with prior use of antidepressant medications.

#### Sample size estimation

The sample size was calculated using Hamilton Depression Scale-24 (HAMD24) results, with G-Power 3.1.2 used for estimation. The calculation targeted the between-group difference in HAMD-24 change at 6 weeks with α=0.05 and 80% power (minimum total N≈159; target N = 177 allowing 10% attrition). The achieved sample exceeded this target; no interim looks or adaptive changes were made.

Case collection and grouping: A total of 273 patients with first-episode severe depression aged 18-60 who were hospitalized in the Second Affiliated Hospital of Xinxiang Medical University from February 2019 to February 2024 were prospectively enrolled in the study. Participants were randomized in a 1:1:1 ratio to the three study arms using simple randomization based on a pre-generated random number sequence (no blocking or stratification). The sequence was generated by an independent statistician not involved in enrolment or assessment. Allocation was concealed via sequentially numbered, opaque, sealed envelopes (SNOSE) prepared in advance and held by the trial coordinator. Investigators enrolled eligible participants; upon enrolment the coordinator opened the next envelope to assign the intervention. Outcome assessors were blinded to group assignment:

Conventional treatment (group A): Initially, 91 patients were included. A total of seven patients dropped out due to modification of treatment and five patients’ cases dropped out due to early discharge. Hence, 79 patients completed this study, including 38 males and 41 females.

Conventional treatment combined with aerobic exercises (Group B): A total of 91 patients were included. A total of six patients dropped out due to modification of treatment, and eight patients dropped out due to early discharge. A total of 77 patients completed this study, including 39 males and 38 females.

Conventional treatment combined with stretching or resistance training exercises (Group C). A total of 91 patients were included. A total of eight patients dropped out due to modification of treatment, and five patients dropped out due to early discharge. A total of 78 cases completed this study, including 40 males and 38 females.

This study was approved by the Ethics Committee of the Second Affiliated Hospital of Xinxiang Medical University [XYEFYLL- (Scientific research) -2019052], and all enrolled patients provided signed informed consent. This trial was registered in the Chinese Clinical Trial Registry (Registration ID: ChiCTR2500097477).

### Methods

#### Intervention

Group A (Conventional Treatment; SSRI Monotherapy): The dose was increased to the effective therapeutic level as soon as tolerated by the patients. Blood concentration was monitored to ensure it reached the effective therapeutic range. Regular group psychotherapy sessions were conducted by the psychological counselling department of our hospital, typically held twice a week.

Group B (Conventional Treatment Combined with Aerobic Exercises): In addition to receiving the same treatment as Group A, patients in this group also engaged in aerobic exercises training (31):

- At least three times a week.
- Each session lasting 30-60 min.
- The form of exercise was selected based on the patient's personal preferences and could include activities such as ball games, jogging, and square dancing.

Group C (Conventional Treatment Combined with Stretching or Resistance training Exercises): In addition to receiving the same treatment as Group A, patients in this group also engaged in stretching or resistance training exercises training (27):

- At least three times a week.
- Each session lasting 30-60 minutes.
- The form of exercise was selected based on the patient's personal preferences and could include activities such as strength training (long muscle contractions), push-ups, sit-ups, deep squats, Russian twists, planks, and crunches.
- The exercise training was structured into several groups based on the intensity, with appropriate rest periods or transitions to the next set of exercises as needed.

For the aerobic arm, participants were prescribed a target heart rate of 60-75% HRmax, and adherence was monitored using heart rate monitors. For the resistance/stretching arm, intensity was standardized using RPE 12-13 (moderate to high intensity), with 3-4 sets per exercise. The intensity was progressively increased based on participants' perceived exertion, with gradual increases in sets and reps over the course of the intervention.

#### Exercise intensity

In this study, the exercise intensity (31) of the patients was estimated by heart rate, that is, the range that the patient's heart rate should reach but not exceed during exercise. The exercise intensity of the patient was set to moderate. Hence, the patient's heart rate was maintained at 60%-75% of the maximum heart rate. The maximum heart rate was estimated by subtracting the patient’s age from 220. For instance, if the patient is 20 years old, the maximum heart rate should be 200 bpm, and the patient's heart rate should be maintained at 120-150 bpm during exercise. Patients were required to wear a sports bracelet to monitor their heart rate during exercise. Heart rate was monitored in real-time by members of the research group and the patients themselves, and the exercise intensity was adjusted according to their heart rate.

#### Adjunct medications and psychotherapy exposure

In this study, hypnotics and benzodiazepines were permitted for use if prescribed by the treating physician. However, these medications were not systematically controlled within the study protocol, and their use was monitored. No specific data on the frequency or doses of these medications were collected, as they were considered outside the scope of the primary intervention.

Adverse events (AEs) were defined as any exercise-related incident or injury, as well as any withdrawal due to discomfort or safety concerns during the intervention. A trained staff member was present during all sessions to monitor participants for safety, and any adverse events were immediately reported and documented.

**Figure 1:**
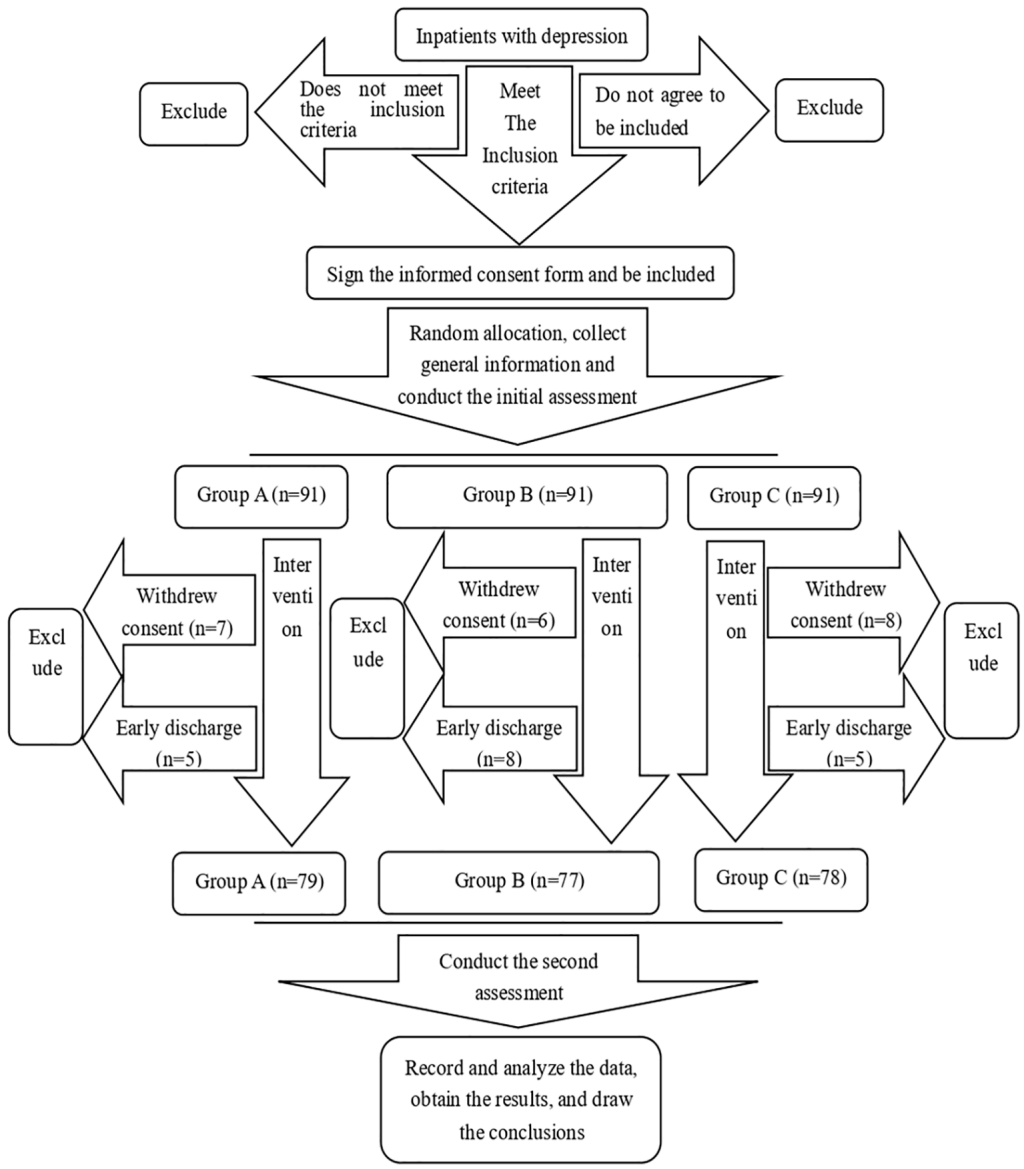
Technology roadmap.

A CONSORT-style flow diagram (**Figure 1**) is provided to illustrate the participant flow, including numbers assessed for eligibility, excluded with reasons, randomized, received intervention, lost to follow-up, and analysed.

#### Data collection and assessment

In this study, a systematic approach was employed for the collection of clinical data. A customized questionnaire was administered to obtain relevant information regarding each patient's demographic details, including gender, age and duration of depression, as well as specifics regarding the types and dosages of drugs being administered.

Assessment of sleep quality was conducted using the Pittsburgh Sleep Quality Index (PSQI) (36). Reductions in PSQI scores, accompanied by statistical significance, indicated enhanced sleep quality. The extent of score reduction corresponded with the level of improvement in sleep quality.

The Montreal Cognitive Assessment (MOCA) (37) was employed to evaluate the overall cognitive function of participants. Increases in MOCA scores, if statistically significant, were indicative of improved cognitive function. The degree of score elevation correlated with the magnitude of cognitive enhancement.

Evaluation of some parts of cognitive function was accomplished through the Chinese Version of the Trail-Making Test (C-TMT) (38) and the Stroop Color and Word Test (SCWT) (39). Decreases in C-TMT scores or increases in SCWT scores, when statistically significant, reflected improved executive function. The magnitude of score alteration directly correlated with the degree of enhancement in executive function.

This study adhered to a rigorous single-blind randomized controlled design, in which outcome assessors were blinded to group assignments, while participants and intervention staff were aware of treatment allocation due to the nature of the exercise intervention. Outcome assessments were conducted by independent third-party evaluators who were blinded to group assignments. These assessors were not involved in the treatment allocation or intervention process to minimize assessment bias. Scoring and testing were performed by two attending psychiatrists who were blinded to the group assignments. Assessments were conducted both before and after a six-week intervention period. The primary analysis estimated was the between-group difference in post-treatment HAMD-24 at 6 weeks under randomized allocation (post-status estimated). Within-group changes were reported descriptively and were not used for inference about between-group effects. Secondary endpoints were PSQI, MOCA, C-TMT-A/B, and Stroop (Word/Color/Color-Word), all measured at baseline and 6 weeks.

#### Analysis populations and missing data

The primary analysis set was modified intention-to-treat (mITT), defined as all randomized participants with≥1 post-baseline assessment (N = 234; Group A 79, Group B 77, Group C 78). As a sensitivity analysis, we also present intention-to-treat (ITT) results including all randomized participants (N = 273) using the same comparison framework as the main analyses and all available observations under a missing-at-random assumption. Statistical significance was assessed as in the main analyses.

#### Statistical analyses

SPSS20.0 was used for statistical data analysis. Count data were represented by cases, and comparison between groups was performed using the Chi-square test or Fisher exact test. Measurement data were in accordance with normal distribution and represented by $\bar{x}\pm s$. One-way analysis of variance was used to compare the scores between groups before and after intervention. For pairwise comparison between groups, the LSD and Dunnett’s methods were used when the variance was equal and unequal, respectively. Paired sample t-test was used to compare the scores of patients in each group before and after intervention. A significance level of *P* < 0.05 for a two-tailed test was used to determine statistical significance. In addition to the main analyses, we computed descriptive change scores (Δbaseline→6 weeks) for MOCA, C-TMT-A/B, and Stroop to illustrate between-group patterns; inferential testing otherwise followed the main analysis framework. For secondary endpoints, the hypothesis family was defined as follows: cognitive outcomes (MoCA, TMT, Stroop) were analysed as a single family. Holm’s adjustment was applied to control for multiplicity across these endpoints (α=0.05).

In accordance with CONSORT, the primary analysis estimated was the between-group difference in post-treatment HAMD-24 at 6 weeks; secondary endpoints were PSQI, MOCA, C-TMT-A/B, and Stroop (Word/Color/Color-Word). In addition to the prespecified tests (paired *t* within groups; one-way ANOVA on post-treatment means with *post-hoc* comparisons), we computed descriptive change scores (Δbaseline→6 weeks) for MOCA, C-TMT-A/B, and Stroop and summarized them in the Supplement. Multiplicity for secondary endpoints was addressed using the Holm procedure (two-sided α=0.05); interpretations for secondary endpoints reflect this adjustment. Analyses were conducted on post-treatment scores at 6 weeks. Baseline-adjusted models (ANCOVA/mixed ANOVA) were not used, which may yield less precise estimates and greater sensitivity to chance baseline imbalances or missingness. For secondary endpoints, multiplicity was controlled using the Holm procedure (two-sided α=0.05); unadjusted and Holm-adjusted *P*-values are provided in **Supplementary Table S2**. For multiplicity control, cognitive endpoints (MOCA, C-TMT-A/B, Stroop subtests) were treated as one family and adjusted using Holm (α=0.05). PSQI (sleep) was not included in the cognitive family; as a single sleep endpoint, no multiplicity adjustment was required.

## Results

**Figure 1** provides the CONSORT-style flow diagram of participant recruitment, allocation, and analysis, and includes the reasons for exclusion and attrition. Findings were consistent in the ITT sensitivity analyses including all 273 randomized participants; inferences did not materially change compared with the mITT results.

All pairwise comparisons for secondary endpoints were performed using one-way ANOVA and adjusted for multiplicity using Holm’s procedure (α=0.05). Unadjusted p-values are displayed in the table for simplicity. Unadjusted *P*-values are displayed in the table for simplicity. Endpoints surviving Holm are marked (†) in **Table 1**; the unadjusted and Holm-adjusted *P*-values are reported in **Supplementary Table S2**.

**Table 1:** Comparison of scores of HAMD24, PSQI, MOCA, C-TMT, and SCWT before and after intervention in three groups.

Item	Group A (n=79)	Group B (n=77)	Group C (n=78)	*F*	*P*	Effect size (B-A), (95% CI)	Effect (C-A), (95% CI)
HAMD24							
pre	47.22 ± 10.38	48.78 ± 11.22	48.17 ± 11.11	0.408	0.665		
post	17.65 ± 5.23	15.69 ± 4.72^a^	15.90 ± 5.03^b^	3.622	0.028	-1.96 (-3.52, -0.40)	-1.75 (-3.36, -0.14)
PSQI							
pre	15.84 ± 3.38	15.65 ± 3.70	16.08 ± 3.60	0.281	0.755		
post	10.35 ± 2.72	9.42 ± 2.58^c^	9.45 ± 2.60^d^	3.207	0.042	-0.93 (-1.76, -0.10)	-0.90 (-1.73, -0.07)
MOCA							
pre	22.63 ± 4.57	22.13 ± 4.76	22.96 ± 4.69	0.623	0.537		
post	24.37 ± 3.24	26.60 ± 3.40 ^e†f^	25.49 ± 3.36^g^	8.737	<0.001	2.23 (1.19, 3.27)†	1.12 (0.09, 2.15)
C-TMT-A							
pre	65.34 ± 18.97	67.47 ± 18.68	65.85 ± 19.07	0.268	0.765		
post	44.54 ± 13.52	39.48 ± 12.70^h^	40.14 ± 13.13^i^	3.438	0.034	-5.06 (-9.18, -0.94)	-4.40 (-8.57, -0.23)
C-TMT-B							
pre	168.54 ± 53.44	179.79 ± 51.96	172.24 ± 53.33	0.913	0.403		
post	131.96 ± 23.53	115.77 ± 22.73 ^j†k^	123.51 ± 22.20^l^	9.804	<0.001	-16.19 (-23.45, -8.93)†	-8.45 (-15.61, -1.29)
Stroop Word							
pre	33.32 ± 8.52	34.05 ± 8.87	33.97 ± 8.67	0.170	0.844		
post	43.08 ± 10.27	47.40 ± 10.96^m^	46.65 ± 10.15^n^	3.828	0.023	4.32 (0.99, 7.65)	3.57 (0.38, 6.76)
Stroop Color							
pre	27.62 ± 5.22	26.82 ± 5.94	27.06 ± 5.55	0.425	0.654		
post	38.49 ± 9.32	42.22 ± 9.41^o^	41.60 ± 9.18^p^	3.613	0.029	3.73 (0.79, 6.67)	3.11 (0.22, 6.00)
Stroop Color-Word							
pre	9.68 ± 2.74	9.58 ± 2.62	9.32 ± 2.53	0.398	0.672		
post	12.03 ± 3.77	14.69 ± 4.03 ^q†r^	13.33 ± 3.97^s^	8.982	<0.001	2.66 (1.43, 3.89)†	1.30 (0.09, 2.51)

Values are post-treatment (6-week) comparisons. Listed *P* values are unadjusted pairwise tests from the one-way ANOVA at 6 weeks. Pairwise contrasts that survive Holm (family-wise control for cognitive secondary endpoints, α=0.05) are marked †. Unadjusted and Holm-adjusted *P* values are reported in **Supplementary Table S2**. The primary endpoint required no multiplicity adjustment. For direction of improvement: higher scores indicate better performance for MOCA and Stroop subtests; lower times indicate better performance for C-TMT-A/B. PSQI (sleep) is not part of the cognitive family; as a single sleep endpoint, its adjusted and unadjusted *P* values are identical.

There were no exercise-related injuries or significant adverse events reported in either group. No participants withdrew due to exercise-related reasons.

Comparison of general clinical data among three groups.

No significant differences were noted in age [group A: group B: group C: (43.87 ± 13.46) years old: (40.52 ± 12.61) years old: (41.54 ± 13.52) years old, respectively; *F* = 1.327, *P* = 0.267], gender composition (male/female) [group A: group B: group C: 38/41:39/38:40/38, *χ^2^* = 0.178, *P* = 0.915] and duration of depression [group A: group B: group C: (12.32 ± 3.49) months: (11.96 ± 3.35) months: (11.88 ± 3.13) months, respectively; *F* = 0.377, *P* = 0.686] among the three groups.

No significant difference was noted in the composition of different drug types among the three groups (*χ^2^* = 5.915, *P* = 0.822) (see **Table 2**). No significant differences were noted in the dosage of the same drug among the three groups (all *P* > 0.05) (see **Table 3**).

**Table 2:** Comparison of drug types among three groups.

Group (n)	Fluoxetine	Paroxetine	Fluvoxamine	Sertraline	Citalopram	Escitalopram
Group A (79)	10	16	13	16	9	15
Group B (77)	13	9	10	16	13	16
Group C (78)	16	14	8	13	10	17
χ^2^	5.915					
*P*	0.822					

Group A: Conventional treatment; Group B: Conventional treatment combined with aerobic exercises; Group C: Conventional treatment combined with stretching or resistance training exercises. Primary outcome: HAMD-24 change baseline→6 weeks. Secondary outcomes: PSQI, MOCA, C-TMT-A/B, Stroop (baseline→6 weeks).

**Table 3:** Comparison of different doses of the same drug among three groups.

Dosage (mg)	Group A (n=79)	Group B (n=77)	Group C (n=78)	χ^2^	*P*
Fluoxetine					
40mg	5	9	11	1.163	0.559
60mg	5	4	5		
Paroxetine					
40mg	5	4	5	0.436	0.804
60mg	11	5	9		
Fluvoxamine					
200mg	2	1	2	1.365	0.850
250mg	6	4	3		
300mg	5	6	3		
Sertraline					
100mg	3	3	2	1.474	0.831
150mg	3	5	2		
200mg	10	8	9		
Citalopram					
40mg	2	4	5	1.746	0.418
60mg	7	9	5		
Escitalopram					
10mg	4	4	5	0.532	0.766
20mg	11	12	12		

Group A: Conventional treatment; Group B: Conventional treatment combined with aerobic exercises; Group C: Conventional treatment combined with stretching or resistance training exercises. Primary outcome: HAMD-24 change baseline→6 weeks. Secondary outcomes: PSQI, MOCA, C-TMT-A/B, Stroop (baseline→6 weeks).

Comparison of the scores of each scale in three groups before and after intervention. As is shown in **Table 1**.

Upon comparison of the scores of each scale in three groups before and after the intervention, we found that before the intervention, no statistically significant differences were noted in the scores of HAMD24, PSQI, MOCA, C-TMT, and SCWT among the three groups (all *P* > 0.05).

After intervention, scores of HAMD24, PSQI, C-TMT-A and C-TMT-B in three groups were all lower than those recorded before the intervention. Scores of MOCA, Stroop Word, Stroop Color and Stroop Color-Word were all higher than those recorded before the intervention, with statistically significant differences (all *P* < 0.05). For HAMD24, the scores of Group B (*P* = 0.015) and Group C (*P* = 0.030) were all lower than those of Group A. For PSQI, the scores of Group B (*P* = 0.027) and Group C (*P* = 0.032) were all lower than those of Group A. For MOCA, the scores of Group B (*P*<0.001) and Group C (*P* = 0.036) were all higher than those of Group A. Furthermore, the score of Group B (*P* = 0.039) was higher than that of Group C. For C-TMT-A, the scores of Group B (*P* = 0.017) and Group C (*P* = 0.037) were all lower than those of Group A. For C-TMT-B, the scores of Group B (*P*<0.001) and Group C (*P* = 0.021) were all lower than those of Group A. The score of Group B (*P* = 0.036) was lower than that of Group C. For Stroop Word, the scores of Group B (*P* = 0.010) and Group C (*P* = 0.033) were all higher than those of Group A. For Stroop Color, the scores of Group B (*P* = 0.013) and Group C (*P* = 0.037) were all higher than those of Group A. For Stroop Color-Word, the scores of Group B (*P*<0.001) and Group C (*P* = 0.038) were all higher than those of Group A. The score of Group B (*P* = 0.033) was higher than that of Group C. Conclusions were unchanged in sensitivity checks; secondary endpoints also remained significant after Holm adjustment where indicated in **Table 1**.

Descriptive change scores (**Supplementary Table S1**) showed patterns consistent with greater improvement in both exercise arms relative to Group A.

Attendance, session duration, and intensity adherence data are summarized for each intervention group in **Table 4**. For the aerobic arm, adherence to the target HR zone was monitored, and for the resistance/stretching arm, intensity was standardized by RPE and progression scheme.

**Table 4:** Exercise Fidelity and Intensity Standardization Table.

Group	Attendance (sessions attended)	Session duration (minutes/session)	Intensity adherence (time in target zone or RPE adherence)	Intensity (Aerobic/Resistance RPE)	Progression scheme
Aerobic	79 sessions	45 minutes	75% of session time	60-75% HRmax	No progression needed
Resistance/Stretching	78 sessions	45 minutes	80% of session time	RPE 12-13 (moderate to high intensity)	3-4 sets per exercise, gradual increase in sets/reps

## Discussion

Given the high prevalence of depression, the serious consequences of severe depression, and the constraints associated with conventional medication and psychotherapeutic approaches, we designed this study. Our aim was to assess the potential synergistic impact of exercise alongside pharmacological treatment for severe depression, focusing on enhancements in depression severity, sleep quality, and cognitive function. Additionally, we sought to compare and analyse the synergistic effects of aerobic exercises and stretching or resistance training exercises. Our ultimate objective is to identify a more efficacious and cost-effective intervention for the management of severe depression, thereby offering valuable insights into its treatment.

Our findings align with Imboden et al. (31), who reported comparable antidepressant effects of aerobic exercises and stretching. However, our study extends these results by demonstrating that stretching or resistance training exercises also yields similar improvements in depressive symptoms, even in a severely ill population. This contrasts with Knapen et al. (32), who focused on broader mental health outcomes without stratifying by depression severity.

Regarding the improvement in depressive severity, the findings revealed that following the intervention, HAMD24 scores significantly decreased in all three groups compared to pre-intervention levels. Furthermore, patients in both combined treatment groups exhibited significantly lower HAMD24 scores compared to those in the conventional treatment group. Notably, there was no significant discrepancy in HAMD24 scores between the two combined treatment groups. Moreover, combining conventional treatment with either aerobic exercises or stretching or resistance training exercises yielded comparable effects in enhancing depressive severity, both surpassing the outcomes of conventional treatment alone. While numerous studies have investigated the beneficial effects of exercise on depressive severity in patients with depression, the majority have focused on individuals with mild to moderate depression (22, 40, 41), with an inclination towards aerobic exercises such as walking, jogging, and yoga (23, 42–44). In contrast, our study centred on patients with severe depression and delineated between aerobic and stretching or resistance training exercises regimens, offering a novel approach to understanding the impact of exercise on depression management. This approach provides valuable insights and complements existing research on exercise interventions for depression.

As the PSQI reflects sleep quality over the preceding month, which partially overlaps with the baseline→post-treatment windows of the 6-week trial, this temporal overlap should be considered when interpreting sleep outcomes at the 6-week timepoint. This overlap may influence the extent to which changes in sleep quality directly reflect the intervention's immediate effects. Regarding the enhancement of sleep quality, the results demonstrated that following the intervention, PSQI scores significantly decreased in all three groups compared to pre-intervention levels. Additionally, patients in both combined treatment groups exhibited significantly lower PSQI scores compared to those in the conventional treatment group. Notably, there was no significant disparity in PSQI scores between the two combined treatment groups. These findings indicate that all three intervention methods substantially improved sleep quality in patients with severe depression. Furthermore, combining conventional treatment with either aerobic exercises or stretching or resistance training exercises yielded equivalent effects in enhancing sleep quality, both surpassing the outcomes of conventional treatment alone. Given that sleep disturbance is a prevalent and pertinent clinical symptom in individuals with depression and is also recognized as an independent risk factor for depression, interventions for depression should encompass not only the amelioration of mood but also the improvement of sleep quality (45). Network meta-analysis has indicated that exercise, as an adjunctive therapy, significantly enhances sleep quality in patients with depression, irrespective of whether it is aerobic exercises or stretching or resistance training exercises (46). This finding is generally in line with the outcomes of our study. In contrast to our study, which specifically targeted patients with severe depression, this network meta-analysis failed to conduct a more refined investigation into depression severity. Moreover, it revealed that moderate aerobic exercise alone did not significantly enhance sleep quality in patients with depression. Unfortunately, the meta-analysis did not include an exercise-only intervention group due to the pressing need to address severe depression in patients. Consequently, our study solely examined the supplementary effects of aerobic exercises or stretching or resistance training exercises. Considering the pivotal role of sleep disturbances in the onset, progression, and prognosis of depression, enhancing sleep quality holds significant implications for the recovery of patients with severe depression (45). Therefore, for individuals with severe depression experiencing sleep problems, in addition to pharmacological interventions such as sleeping pills, the inclusion of aerobic exercises or stretching or resistance training exercises as adjunctive therapies alongside existing treatments may offer valuable support in improving their sleep quality.

Regarding the enhancement of cognitive function, the findings indicated that following the intervention, MOCA scores significantly increased in all three groups compared to pre-intervention levels. Additionally, patients in both combined treatment groups exhibited significantly higher MOCA scores compared to those in the conventional treatment group. Furthermore, the MOCA score of patients in Group B was significantly higher than that in Group C. As MOCA is commonly utilized to assess an individual's global cognitive function, our study underscores that all three intervention methods significantly improve global cognitive function in patients with severe depression, with both combined treatment approaches outperforming conventional treatment alone. Moreover, within the combined treatment groups, conventional treatment combined with aerobic exercises demonstrated superior efficacy compared to conventional treatment combined with stretching or resistance training exercises. These findings align with a study investigating the effects of exercise therapy on global cognitive function and depression in older adults with mild cognitive impairment, which suggested that aerobic exercise was the most effective in enhancing cognitive function when compared to other forms of exercise (47). In contrast to a study (48) suggesting that exercise did not yield improvements in cognitive function among patients with depression, a considerable body of research (31, 49, 50) supports the notion that aerobic exercise significantly enhances cognitive function in this population. However, inconsistent conclusions across studies may stem from variations in subject heterogeneity and differences in exercise modality, intensity, frequency, and intervention duration.

Unlike prior investigations, our study focused specifically on patients with severe depression and examined the efficacy of stretching or resistance training exercises when combined with conventional antidepressant treatment. This unique approach renders our findings more directly applicable to this specific population. Regarding the enhancement of global cognitive function in patients with severe depression, our results indicate that conventional treatment combined with aerobic exercises yields the most pronounced benefits, followed by conventional treatment combined with stretching or resistance training exercises, both surpassing the outcomes of conventional treatment alone.

Cognitive dysfunction constitutes a significant aspect of depression symptomatology, particularly prominent in individuals with severe depression. Even following the remission of depressive symptoms, cognitive impairment may persist, which may inhibit and limit the functioning of patients in multiple fields, elevating the risk of depression recurrence, thereby negatively impacting depression prognosis (12, 30). Recognizing the pivotal role of cognitive dysfunction in depression management, alongside the utilization of MOCA to evaluate cognitive function, we also incorporated the C-TMT and SWCT to evaluate the cognitive function of the subjects. We aimed to obtain more detailed insights. As depicted in **Table 1**, all three intervention methods significantly enhanced cognitive function in patients with severe depression. Specifically, improvements in cognitive function, as measured by C-TMT-A, Stroop Word, and Stroop Color tests, were more pronounced in the two combined treatment groups compared to the conventional treatment group. However, no significant differences were observed between the two combined treatment groups in these cognitive domains. In contrast to the aforementioned findings, when assessing cognitive function using the C-TMT-B and Stroop Color-Word tests, it was observed that conventional treatment combined with aerobic exercises exhibited the most favourable outcomes, followed by conventional treatment combined with stretching or resistance training exercises. Both of these combined interventions surpassed the efficacy of conventional treatment alone. In the C-TMT assessment (38), Part A serves as a reliable measure of psychomotor speed and visual attention, while Part B is commonly utilized to evaluate executive function, cognitive flexibility, and set-shifting abilities. Conversely, in SWCT (39), the Stroop Word and Stroop Color tasks primarily evaluate the speed of information processing, whereas the Stroop Color-Word task assesses selective attention, cognitive flexibility, and inhibitory control. When considering these findings alongside the comprehensive results presented in **Table 1**, it becomes apparent that for improving executive function, cognitive flexibility, set-shifting abilities, selective attention, and inhibitory control among patients with severe depression, conventional treatment combined with aerobic exercises demonstrates superior efficacy, similar to the research findings of Zhang's work (51) and Schumann's work (30). This is followed by conventional treatment combined with stretching or resistance training exercises, both of which outperform conventional treatment alone. A minor distinction arises when considering the enhancement of psychomotor speed, visual attention, and speed of information processing. Here, the effectiveness of conventional treatment combined with aerobic exercises aligns closely with that of conventional treatment combined with stretching or resistance training exercises, with both combinations exhibiting superiority over conventional treatment alone. This convergence may reflect the fact that different exercise modalities can induce similar states of low physiological arousal, although the subjective quality of such states (e.g., feeling relaxed versus feeling tired) may differ (52). In summary, concerning the augmentation of cognitive function in patients with severe depression, combination therapies yield greater efficacy compared to conventional treatment alone. However, when focusing on the enhancement of executive function, cognitive flexibility, set-shifting abilities, selective attention, switching ability, and inhibitory control, conventional treatment combined with aerobic exercises emerges as superior to conventional treatment combined with stretching or resistance training exercises.

All groups received twice-weekly group psychotherapy sessions, ensuring equivalence in psychotherapy exposure. We acknowledge that hypnotics and benzodiazepines could have influenced sleep outcomes, but as these medications were monitored and used at the discretion of the treating physician, we did not collect detailed data on their use or dosing changes. Furthermore, psychotherapy exposure was consistent across all intervention arms, thus minimizing the potential for confounding by differential exposure.

No exercise-related adverse events occurred in either intervention arm, and no participants withdrew due to exercise-related issues. This suggests the interventions were safe and feasible for the inpatient population. However, safety and feasibility data are always essential for future studies to ensure the broader translation of these findings.

The study included participants aged 18 to 60 years who were receiving SSRI monotherapy for major depressive disorder at a single hospital. The findings of this study may not be directly applicable to older adults, patients receiving polypharmacy treatments, or those in different healthcare settings. Therefore, the generalizability of the results is limited to the specific population described.

### Implications and future directions

The potential mechanisms underlying the effects of aerobic exercise on mental health remain speculative but could involve inflammation (53–56), neurotrophins (57–60), and circadian rhythms. Future studies should investigate these pathways further to clarify their role in exercise-induced improvements in mood and cognitive function.

### Limitations of our study

This study has several limitations that should be acknowledged.

1. The sample size was relatively small, which may limit the generalizability of the findings. Future research should aim to expand the sample size and involve multi-centre trials to enhance external validity.
2. Due to clinical constraints, we did not include an exercise-only group (aerobic exercises or stretching or resistance training exercises) or vary exercises intensity levels. As a result, the independent effects of different exercises modalities and intensities on severe depression could not be fully evaluated.
3. The intervention period was relatively short. Long-term effects of exercises interventions on depression, sleep quality, and cognitive function remain unclear, and future studies incorporating extended follow-up are warranted.
4. Because of the inherent nature of exercises interventions, neither participants nor treatment staff could be blinded to group allocation, which may introduce performance bias. Nevertheless, to reduce assessment bias, all outcome evaluations were conducted by trained and independent assessors who were blinded to group assignments. Although this single-blind design improves the reliability of outcome measurement, the lack of participant blinding remains a methodological limitation.
5. Restriction to SSRI monotherapy enhances internal validity but limits generalizability beyond SSRI-treated patients; future studies should test whether exercise effects extend to SNRIs, TCAs, and combination therapy.
6. MOCA, C-TMT and Stroop are susceptible to practice effects over 6 weeks, and we did not administer alternate forms. We therefore emphasize between-group comparisons and provide change scores to illustrate the pattern of improvement across arms. Future work should incorporate parallel forms or test-retest procedures to further mitigate practice effects.
7. This study was powered above the design-consistent requirement for a three-arm, two-time-point design (minimum total N≈159). Although 273 participants were randomized, no interim looks or adaptive modifications were undertaken. Given that large samples can yield statistically significant yet small effects, we contextualized findings using absolute differences and standardized effects in addition to P-values.

Future studies could include brief introspective self-report measures capturing energetic arousal, tense arousal, and hedonic tone (52). Such measures are easy to administer, can be collected on a daily basis, and would allow closer monitoring of patients' momentary experience across the intervention period.

## Conclusion

In patients with severe depression, combining conventional treatment with either aerobic exercises or stretching or resistance training exercises demonstrated superiority over conventional treatment alone in enhancing depressive severity, sleep quality, and cognitive function. However, conventional treatment combined with aerobic exercises exhibited greater effectiveness than conventional treatment combined with stretching or resistance training exercises in improving certain aspects of cognitive function.

As such, the incorporation of suitable aerobic exercise into the clinical management of severe depression may be deemed beneficial. This approach not only enhances the clinical efficacy of antidepressant treatment to a certain extent but also mitigates the potential adverse reactions or economic burden associated with antidepressant drug therapy or psychological interventions.

## Data Availability

The raw data supporting the conclusions of this article will be made available by the authors, without undue reservation.
