# Supplementary material for: Effects of exercise supplementary to standard therapy on cognition and sleep in depression: a randomised controlled trial
Source: Front Psychiatry. 2026 Feb 3;17:1650334. doi: 10.3389/fpsyt.2026.1650334 (PMC12909528; doi:10.3389/fpsyt.2026.1650334)
Supplement: Supplementary file 1 [file Table1.doc]

Descriptive change scores (Δ baseline→6 weeks) for cognitive outcomes

| Outcome | ΔA (mean) | ΔB (mean) | ΔC (mean) | Δ(B−A) | Δ(C−A) |
| --- | --- | --- | --- | --- | --- |
| MOCA | +1.74 | +4.47 | +2.53 | +2.73 | +0.79 |
| C-TMT-A | -20.80 | -27.99 | -25.71 | -7.19 | -4.91 |
| C-TMT-B | -36.58 | -64.02 | -48.73 | -27.44 | -12.15 |
| Stroop Word | +9.76 | +13.35 | +12.68 | +3.59 | +2.92 |
| Stroop Color | +10.87 | +15.40 | +14.54 | +4.53 | +3.67 |
| Stroop Color-Word | +2.35 | +5.11 | +4.01 | +2.76 | +1.66 |

Δ= post-intervention minus baseline. For C-TMT-A/B, more negative values indicate improvement (shorter time). No inferential tests are presented here; see main text for between-group comparisons.
