# Supplementary material for: Effects of exercise supplementary to standard therapy on cognition and sleep in depression: a randomised controlled trial
Source: Front Psychiatry. 2026 Feb 3;17:1650334. doi: 10.3389/fpsyt.2026.1650334 (PMC12909528; doi:10.3389/fpsyt.2026.1650334)
Supplement: Supplementary file 2 [file Table2.doc]

Supplementary Table S2

| **Endpoint (cognitive family)** | **Contrast** | **Unadjusted *P*** | **Holm-adjusted *P*** | **Survives Holm** |
| --- | --- | --- | --- | --- |
| **MOCA** | **B vs A** (e) | <0.001 | **0.015** | **†** |
| MOCA | C vs A (g) | 0.036 | 0.264 |  |
| MOCA | B vs C (f) | 0.039 | 0.264 |  |
| **C-TMT-A** | B vs A (h) | 0.017 | 0.170 |  |
| C-TMT-A | C vs A (i) | 0.037 | 0.264 |  |
| **C-TMT-B** | **B vs A** (j) | <0.001 | **0.015** | **†** |
| C-TMT-B | B vs C (k) | 0.036 | 0.264 |  |
| C-TMT-B | C vs A (l) | 0.021 | 0.189 |  |
| **Stroop Word** | B vs A (m) | 0.010 | 0.120 |  |
| Stroop Word | C vs A (n) | 0.033 | 0.264 |  |
| **Stroop Color** | B vs A (o) | 0.013 | 0.143 |  |
| Stroop Color | C vs A (p) | 0.037 | 0.264 |  |
| **Stroop Color-Word** | **B vs A** (q) | <0.001 | **0.015** | **†** |
| Stroop Color-Word | B vs C (r) | 0.033 | 0.264 |  |
| Stroop Color-Word | C vs A (s) | 0.038 | 0.264 |  |
